# Screen use and consumption of ultra-processed foods among Brazilian adolescents

**DOI:** 10.11606/s1518-8787.2026060007360

**Published:** 2026-09-09

**Authors:** Maria Eduarda da Costa Andrade, Diôgo Vale, Poliana de Araújo Palmeira

**Affiliations:** I Universidade Federal da Paraíba Programa de Pós-Graduação em Ciências da Nutrição João Pessoa PB Brasil Universidade Federal da Paraíba. Programa de Pós-Graduação em Ciências da Nutrição. João Pessoa, PB, Brasil; II Instituto Federal de Educação, Ciência e Tecnologia do Rio Grande do Norte Jucurutu RN Brasil Instituto Federal de Educação, Ciência e Tecnologia do Rio Grande do Norte. Jucurutu, RN, Brasil

**Keywords:** Adolescent, Processed Foods, Screen Time, Food and Nutritional Surveillance, Health Surveys

## Abstract

**OBJECTIVE:**

To investigate the association between screen time, including time spent watching television and using media (television, computer, and cell phone) during meals, and high consumption of ultra-processed foods among Brazilian adolescents.

**METHODS:**

A population-based cross-sectional study using data from the 2019 Pesquisa Nacional de Saúde do Escolar (National School Health Survey), analyzing a sample of 123,761 adolescents (ages 13–17). Consumption of ultra-processed foods was assessed using a score based on the frequency of consumption of thirteen subgroups of ultra-processed foods on the previous day. High consumption was defined as a score of seven or higher, corresponding to the top quintile. The association was investigated using Poisson regression models, adjusted for sex, age, self-reported race/skin color, geographic region, location (urban/rural), school type (public/private), and wealth index.

**RESULTS:**

The prevalence of high consumption of ultra-processed foods (scores ≥ 7) was 16.15% (95% CI 15.46–16.86). A dose-response relationship was observed, with higher prevalences associated with increased screen time, both for time spent watching television and for media use during meals. Adolescents who watched television for > 6 h/day had a 90% higher prevalence of high consumption of ultra-processed foods (PR_a_ = 1.90; 95% CI 1.72–2.11) compared with those who did not watch television. The association was significant even in the category with the lowest exposure time (up to 1 h/day). Similarly, adolescents who used media every day during meals had a 55% higher prevalence (PR_a_ = 1.55; 95% CI 1.43–1.68).

**CONCLUSIONS:**

The findings demonstrate a direct and significant association between increased screen time and high consumption of ultra-processed foods among Brazilian adolescents. This study reinforces the need for public health interventions that address excessive screen time as a key behavioral factor in the consumption of ultra-processed foods in this population.

## INTRODUCTION

Ultra-processed foods (UPF) are industrially produced formulations rich in additives and food-derived substances, containing little or no fresh foods. They include sugars, oils, fats, or salt, as well as other components not typically found in culinary preparations, such as hydrogenated oils and corn syrup^1^. Their ingredients also include cosmetic food additives, which modify the product’s sensory characteristics, such as flavorings, colorings, sweeteners, thickeners, and flavor enhancers^2,3^.

Globally, consumption of UPF is higher among younger age groups. In the United Kingdom and the United States, for example, UPF account, on average, for 68.0% and 67.0% of daily energy intake among adolescents, respectively^4, 5^. Although consumption is comparatively lower in low- and middle-income countries, in Chile, Colombia, and Brazil, adolescents are among the heaviest consumers of these products^6^. In Brazil, specifically, UPF accounted for approximately 26.5% of the total energy consumed by adolescents between 2017 and 2018^8^.

Pesquisas de Orçamentos Familiares (Household Budget Surveys) indicated systematic increases in the purchase of all major categories of UPF by Brazilian households, accompanied by a reduction in the purchase of fresh and minimally processed foods. These trends in changing dietary patterns, marked by the growing consumption of UPF, are consistent with the rising prevalence of chronic noncommunicable diseases, as well as overweight and obesity in the population^9^. In 2019, 19.4% of Brazilian adolescents were overweight, with obesity affecting 8.0% of girls and 5.4% of boys^10^.

Studies have demonstrated an association between an increase in the dietary proportion of UPF and higher energy density, greater amounts of free sugars, and lower dietary fiber content. These findings suggest that UPF consumption is a potential determinant of obesity in adolescents. Consistent consumption of UPF over time negatively impacts nutritional status and body composition in this age group^11^. Given the limited progress in reducing chronic noncommunicable diseasesamong adolescents and the increase in obesity-related diseases^12^, it is urgent to identify their main risk factors.

Rocha et al.^13^ found, based on a cross-sectional analysis of data from the 2013 and 2014 Estudo de Riscos Cardiovasculares em Adolescentes (Study on Cardiovascular Risks in Adolescents), that the percentage of caloric intake from UPF among Brazilian adolescents was significantly higher among those who attended private schools, did not eat the meals provided by schools, did not eat breakfast regularly, ate while watching television (TV) every day or almost every day, and spent more time in front of screens. Consistent with this, another recent national study^14^ observed an association between excessive use of TV and video games and poorer diet quality among adolescents, as assessed by the weekly frequency of dietary intake markers.

Excessive screen use is another determinant of overweight in adolescents^12^, attributed primarily to changes in diet quality^15^. Until recently, TV was the most common form of screen time among adolescents, but there is currently an increase in the use of other types of screens, such as computers and cell phones, especially during meals. These habits may affect food intake and need to be further investigated^16^. Although the relationship between UPF and screen time has been explored individually, the association between TV time, frequency of using different media during meals, and UPF consumption among adolescents has not yet been sufficiently investigated in nationally representative samples.

Understanding this complex relationship between UPF consumption and screen time is essential for developing effective public health strategies to prevent obesity and chronic diseases. This understanding is necessary for promoting adequate and healthy nutrition and for ensuring the human right to adequate food. Furthermore, the Guia Alimentar para a População Brasileira (Dietary Guidelines for the Brazilian Population)^17^ recommend the consumption of fresh and minimally processed foods, discouraging the consumption of processed foods. In this context, the objective of this study was to investigate the association between time spent watching TV, media use during meals, and the consumption of UPF in a nationally representative sample of Brazilian adolescents aged 13 to 17 years.

## METHODS

### Study Design and Population

This cross-sectional study was conducted using data from the 2019 Pesquisa Nacional de Saúde do Escolar (PeNSE – National School Health Survey), a nationwide school-based survey conducted in Brazil by the Instituto Brasileiro de Geografia e Estatística (IBGE – Brazilian Institute of Geography and Statistics), in partnership with the Ministry of Health and the Ministry of Education. The objective of the survey is to collect information on the health of adolescents aged 13 to 17 who attend public and private schools in urban and rural areas throughout the country^18^.

The PeNSE 2019 sampling plan used two-stage cluster sampling, involving schools and classes, which allowed for the estimation of population parameters for Brazil, the Major Regions, the Federal Units, and the state capitals^14^. A total of 124,620 adolescents were included. Data was collected from April to September 2019 in classrooms using self-administered questionnaires on mobile data-collection devices, under supervision^18^. After excluding 849 students with missing data on UPF use, the final sample for these analyses comprised 123,761 participants. Considering the effect of the sampling design, the data represent 11,735,113 Brazilian adolescents. For the association analyses (regression models), the analytical sample consisted of 101,923 and 101,998 participants, respectively, for the exposures “time spent watching TV” and “media use during meals.” This reduction compared to the final sample is due to the exclusion of missing data for any of the exposure variables and covariates included in the models.

### Outcome Variable

Consumption of processed foods was assessed using a questionnaire (yes or no) regarding the consumption of 13 subgroups of processed foods on the day prior to the interview. These subgroups included: (1) soda; (2) fruit juice in a carton or can; (3) powdered soft drink; (4) chocolate-flavored beverage; (5) flavored yogurt; (6) packaged snacks (chips) or salty cookies/crackers; (7) sweet cookies/crackers, filled cookies, or packaged cookies; (8) chocolate, ice cream, gelatin, flan*,* or other processed desserts; (9) sausage, hot dog, bologna, or ham; (10) sliced bread, hot dog buns, or hamburger buns; (11) margarine; (12) mayonnaise, ketchup*,* or other processed sauces; and (13) instant noodles, instant soup, frozen lasagna, or other frozen ready-to-eat meals. An UPF consumption score was created by summing the number of affirmative responses, ranging from 0 (did not consume any subgroup) to 13 (consumed all subgroups). The outcome (high UPF consumption) was defined as scores of 7 or higher. This cutoff corresponds to the (approximate) top quintile of the score distribution in the sample and is consistent with previous epidemiological studies^19^.

### Exposure Variables

TV viewing time was reported by the adolescents, considering only weekdays (excluding Saturdays, Sundays, and holidays) and six response categories: does not watch, up to 1 h/day, > 1 h – 2 h/day, > 2 h – 3 h/day, > 3 h – 6 h/day, and > 6 h/day.

The frequency of media use during meals (TV, computer, or cell phone) was assessed using five response categories: none, one to two days, three to four days, five to six days, and every day.

### Covariates

The covariates investigated included the participants’ sociodemographic characteristics: sex (male and female), age (13 –15 years, 16 – 17 years), self-reported race/skin color (white, brown, black, yellow, and Indigenous), geographic region (North, Northeast, Southeast, South, and Central-West of Brazil), location (urban and rural), and school administrative status (public or private). Socioeconomic status was assessed using a wealth index adapted from Barros and Victora^22^, constructed using principal component analysis (PCA). The variables included in the PCA were: ownership of a cell phone, computer/laptop, and car; internet access; number of bathrooms in the household (0, 1, 2, and 3 or more); presence of a domestic worker; and the mother’s educational level (incomplete elementary school, complete elementary school, incomplete high school, complete high school, incomplete college, and complete college). The first principal component, which explains the largest proportion of the total variance in the data, was retained to create a continuous measure of wealth. This variable was categorized into quintiles, with the first quintile (Q1) representing the 20% of adolescents with the lowest wealth and the fifth quintile (Q5) representing the 20% with the highest wealth.

### Data Analysis

The sociodemographic characteristics of the population were described using relative frequencies (%) and their respective 95% confidence intervals (95% CI), both for the total population and stratified by outcome (high UPF consumption) and by exposures (TV time and media use during meals). Differences in prevalence between sociodemographic categories and exposures were assessed by the overlap of the 95% CI; a lack of overlap indicated statistical significance. Next, the prevalence of high UPF consumption (≥ 7 subgroups) among adolescents was described according to TV time and frequency of media use during meals.

To investigate the association between each exposure (TV time and frequency of media use during meals) and high UPF consumption, Poisson regression models with robust variance were employed. Crude and adjusted prevalence ratios (PR) were estimated, along with their respective 95% CI. The exposure variables were also included as ordinal variables in the models to examine the linear relationship with high UPF consumption. All adjusted models were controlled for potential confounding factors, including sex, age, race/skin color, wealth index, region of the country, school location (urban/rural), and school administrative unit (public/private).

All statistical analyses were performed using Stata version 15, applying the svy command to incorporate the PeNSE 2019 weighting factors and the two-stage complex sampling design, ensuring the representativeness of the study population. Statistical significance was defined *a priori* by a 95% CI that did not contain the value one and by a p-value ≤ 0.05.

### Ethical Considerations

Student participation was voluntary and contingent upon signing the Informed Consent Form, which appeared on the first screen of the PeNSE 2019 app on the mobile data collection device. Access to the questionnaire was granted only after confirmation of this consent. The survey was approved by the National Research Ethics Commission of the National Health Council (Opinion No. 3,249,268)^18^.

## RESULTS

The final sample consisted of 123,761 Brazilian adolescents, with a similar distribution by sex. Most participants (64.70%) were between 13 and 15 years old and self-identified as black, that is, mixed-race (43.57%) or black (13.52%). More than one-third of the sample belonged to the poorest quintile of the wealth index (31.81%), and the majority lived in urban areas (92.42%) and attended public schools (85.46%) (Table 1).

**Table 1:** Sociodemographic characteristics, consumption of ultra-processed foods, and screen time among Brazilian adolescents. PeNSE, 2019 (n = 123,761).

Variable	Total	
	%	95% CI
Gender		
Male	49.14	48.34–49.94
Female	50.86	50.06–51.66
Age group		
13–15 years	64.70	62.39–66.95
16–17 years	35.30	33.05–37.61
Race/skin color		
White	36.06	35.21–36.91
Brown	43.57	42.71–44.44
Black	13.52	12.99–14.06
Yellow	3.73	3.49–3.99
Indigenous	3.12	2.89–3.37
Wealth Index		
1st (poorest)	31.81	30.57–33.07
2nd	25.09	24.37–25.82
3rd	21.23	20.45–22.03
4th	14.41	13.75–15.09
5th (richest)	7.47	7.06–7.89
Region		
North	10.81	9.94–11.75
Northeast	28.40	27.20–29.62
Southeast	38.76	37.22–40.34
South	13.75	12.93–14.62
Central-West	8.27	7.80–8.78
Location		
Rural	7.58	6.36–9.01
Urban	92.42	90.99–93.64
School administrative unit		
Public	85.46	84.67–86.21
Private	14.54	13.79–15.33
UPF consumption		
< 7 subgroups	83.85	83.14–84.54
≥ 7 subgroups	16.15	15.46–16.86
Time spent watching TV		
Does not watch	23.21	22.57–23.86
Up to 1 hour/day	24.84	24.28–25.41
> 1–2 hours/day	15.89	15.47–16.32
> 2–3 hours/day	11.00	10.61–11.40
> 3–6 hours/day	15.78	15.31–16.26
> 6 hours/day	9.28	8.84–9.73
Media use during meals		
None	27.91	27.15–28.70
1 to 2 days	7.95	7.60–8.33
3 to 4 days	7.21	6.90–7.55
5 to 6 days	6.07	5.80–6.37
Every day	50.84	49.91–51.78

PeNSE: Pesquisa Nacional de Saúde do Escolar (National School Health Survey); 95% CI: 95% confidence interval; UPF: ultra-processed foods.

Note: missing data: race/skin color (n = 2,462), wealth index (n = 19,662), time spent watching TV (n = 216), media use during meals (n = 85).

The distribution of the UPF consumption score—which corresponds to the number of subgroups consumed on the day before the interview—ranged from 0 to 13. The scores corresponding to 2 (11.73%), 3 (16.34%), 4 (18.08%), 5 (16.32%), and 6 (12.31%) were the most frequent; 6.42% had a score of 1, and 2.66% had a score of 0 (data not shown). The prevalence of high UPF consumption, defined as the consumption of seven or more subgroups (approximate top quintile), was 16.15% (Table 1).

Approximately four out of every ten adolescents reported excessive TV viewing time (> 2 h/day), with 9.28% reporting watching TV for more than 6 h/day. In addition, about six out of every ten adolescents reported using media (TV, computer, or cell phone) during meals every day (Table 1).

The prevalence of high UPF consumption varied significantly across sociodemographic characteristics. The highest prevalences were observed among younger adolescents (17.10%), those who self-identified as black (18.24%), public school students (16.54%), and those living in urban areas (16.46%). In addition, the prevalence of high UPF consumption was lower in the lowest wealth quintile (13.26%) (Table 2).

**Table 2:** Prevalence (%) and 95% CI of ultra-processed food consumption scores of seven or higher on the day before the interview, according to sociodemographic characteristics. PeNSE, 2019 (n = 123,761).

Variable	Consumption of seven or more subgroups of UPF	
	%	95% CI
Gender		
Male	16.70	15.97–17.46
Female	15.61	14.77–16.48
Age group		
13–15 years	17.10	16.19–18.05
16–17 years	14.40	13.50–15.35
Race/skin color		
White	16.18	15.23–17.17
Mixed	15.68	14.76–16.65
Black	18.24	16.94–19.61
Yellow	15.09	12.95–17.50
Indigenous	15.52	13.67–17.57
Wealth Index		
1st (poorest)	13.26	12.35–14.22
2nd	17.58	16.35–18.88
3rd	18.05	16.80–19.37
4th	19.22	17.80–20.73
5th (richest)	17.98	16.50–19.56
Region		
North	13.84	12.95–14.77
Northeast	14.48	13.60–15.39
Southeast	17.58	16.07–19.19
South	17.72	16.56–18.95
Central-West	15.56	14.46–16.72
Location		
Rural	12.29	10.79–13.96
Urban	16.46	15.73–17.22
School’s administrative affiliation		
Public	16.54	15.74–17.36
Private	13.85	13.29–14.44

PeNSE: Pesquisa Nacional de Saúde do Escolar (National School Health Survey); 95% CI: 95% confidence interval; UPF: ultra-processed foods.

Note: missing data: race/skin color (n = 2,462), wealth index (n = 19,662).

Analysis of screen use also revealed significant sociodemographic disparities. Excessive TV watching (> 2 h/day) was more prevalent among younger adolescents (38.68%) and those in the lowest wealth stratum (40.02%) compared with those in the highest wealth stratum (28.56%). Daily screen use during meals was more common in urban areas (52.06% *versus* 35.95% in rural areas) and among the lowest-wealth group (47.32%) than among the wealthiest (34.98%) (Table 3).

**Table 3:** Prevalence of excessive TV watching (> 2 h/day) (n = 123,545) and daily media use during meals (n = 123,676) among adolescents, by sociodemographic characteristics. PeNSE, 2019.

Variable	Excessive TV viewing (> 2 h/day)		Daily media use during meals	
	%	95% CI	%	95% CI
Gender				
Male	35.58	34.55–36.63	51.52	50.34–52.70
Female	36.51	35.57–37.46	50.19	49.15–51.23
Age group				
13–15 years	38.68	37.79–39.57	50.18	48.99–51.38
16–17 years	31.25	30.09–32.43	52.05	50.71–53.40
Race/skin color				
White	31.91	30.86–32.97	49.04	47.79–50.28
Brown	37.61	36.52–38.71	50.47	49.26–51.69
Black	40.85	38.76–42.98	58.09	56.39–59.78
Yellow	39.79	36.88–42.78	47.81	44.80–50.84
Indigenous	38.03	35.00–41.15	50.47	46.95–53.99
Wealth Index				
1st (poorest)	40.02	38.67–41.39	47.93	46.39–49.47
2nd	36.73	35.33–38.15	54.65	53.12–56.17
3rd	33.34	31.86–34.87	52.88	51.33–54.43
4th	30.98	29.21–32.81	43.71	42.29–45.13
5th (richest)	28.56	26.76–30.44	34.98	33.38–36.62
Region				
North	38.98	37.61–40.38	38.14	35.82–40.50
Northeast	35.05	33.83–36.30	48.53	47.21–49.85
Southeast	37.27	35.76–38.81	57.51	55.69–59.30
South	32.86	30.67–35.15	45.20	43.10–47.31
Central-West	35.26	33.65–36.91	53.57	51.92–55.22
Location				
Rural	38.53	36.10–41.03	35.95	33.30–38.70
Urban	35.85	35.02–36.69	52.06	51.08–53.05
School’s administrative affiliation				
Public	37.35	36.45–38.26	51.92	50.84–53.00
Private	28.67	27.66–29.71	44.51	43.58–45.45

PeNSE: Pesquisa Nacional de Saúde do Escolar (National School Health Survey); 95% CI: 95% confidence interval.

Note: Missing data for excessive TV time: race/skin color (n = 2,453), wealth index (n = 19,576); missing data for daily media use during meals: race/skin color (n = 2,453), wealth index (n = 19,576).

Increased UPF consumption among adolescents was directly linked to longer TV viewing time and frequent screen use during meals (Figure). The prevalence of high UPF consumption was 10 percentage points higher among those who watched more than six hours of TV (22.74%) than among those who did not watch TV (12.54%). Similarly, screen use during all meals raised the prevalence to 18.87%, nearly seven percentage points higher than the prevalence observed among adolescents who did not use screens (11.94%).

**Figure:**
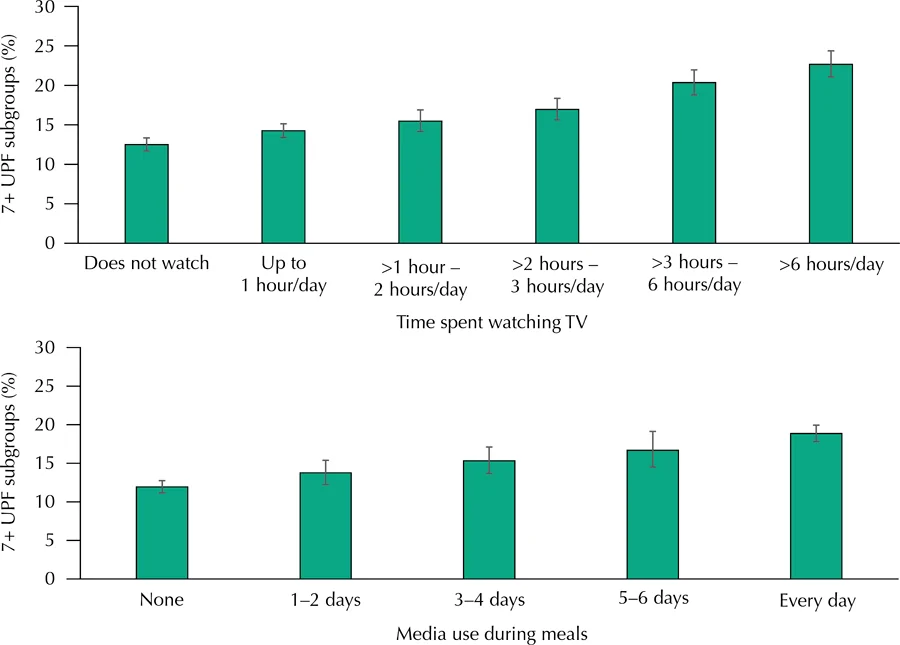
Consumption of seven or more subgroups of ultra-processed foods according to time spent watching television (TV) (n = 123,545) and media use during meals (n = 123,676). PeNSE, 2019.

PeNSE: Pesquisa Nacional de Saúde do Escolar (National School Health Survey); UPF: ultra-processed foods.

As shown in Table 4, the Poisson regression models confirm that both TV watching time and media use during meals are positively associated with high consumption of UPF, independent of all sociodemographic variables analyzed. A dose-response relationship was observed for TV viewing time (trend p < 0.001). Compared with adolescents who did not watch TV, those who watched TV for more than 6 h/day had an adjusted prevalence ratio (PR_a_) of 1.90 (95% CI 1.72–2.11) for high UPF consumption. The association remained significant even for the shortest duration of exposure (up to 1 hour per day), which showed a PR_a_ of 1.17 (95% CI 1.07–1.29).

**Table 4:** Crude and adjusted prevalence ratios for consumption of seven or more subgroups of ultra-processed foods, according to time spent watching TV (n = 101,923) and media use during meals (n = 101,998). PeNSE, 2019.

Variable	Consumption of seven or more subgroups of ultra-processed foods			
	OR	95% CI	Adjusted PR^a^	95% CI
Time spent watching TV				
Does not watch	Ref.	Ref.	Ref.	Ref.
Up to 1 hour/day	1.14	1.05–1.24	1.17	1.07–1.29
> 1–2 hours per day	1.24	1.13–1.36	1.29	1.17–1.44
> 2–3 hours/day	1.36	1.24–1.49	1.41	1.27–1.58
> 3–6 hours/day	1.63	1.49–1.78	1.63	1.47–1.80
> 6 hours/day	1.81	1.65–1.99	1.90	1.72–2.11
Trend p-value			< 0.001	
Media use during meals				
None	Ref.	Ref.	Ref.	Ref.
1 to 2 days	1.15	1.02–1.30	1.16	1.02–1.32
3 to 4 days	1.28	1.14–1.45	1.27	1.11–1.45
5 to 6 days	1.40	1.22–1.60	1.41	1.22–1.64
Every day	1.58	1.46–1.70	1.55	1.43–1.68
Trend p-value			< 0.001	

PeNSE: Pesquisa Nacional de Saúde do Escolar (National School Health Survey); 95% CI: 95% confidence interval; PR: prevalence ratio; UPF: ultra-processed foods.

Note: Poisson regression models were used to assess crude and adjusted associations.

^a^Adjusted for: sex, age group, race/skin color, wealth index, region, location, and school type (public and private).

Similarly, media use during meals showed a positive and significant dose-response relationship with high UPF consumption (trend p < 0.001). Adolescents who used media every day during meals had an PR_a_ of 1.55 (95% CI 1.43–1.68) for high UPF consumption, compared with those who did not use media. The associations were significant starting at one to two days per week (PR_a_= 1.16; 95% CI 1.02– 1.32).

## DISCUSSION

This large cross-sectional study revealed that both time spent watching TV and the use of different types of media during meals were directly associated with high UPF consumption on the day prior to the interview, showing a clear dose-response pattern. The findings highlight the consistent relationship between screen-use behavior and UPF consumption among adolescents.

The results are consistent with previous studies that investigated the relationship between screen exposure and poorer diet quality among adolescents^13,14^. In Brazil, a recent study demonstrated a positive association between time spent watching TV and using other screens and the consumption of UPF^21^. This trend appears to be global, with studies in Chile^16^ and the United Kingdom^4^ reinforcing the link between increased screen time and poorer-quality diets. Furthermore, a systematic review^15^ found that the habit of eating while watching TV among children and adolescents is linked to higher intake of energy-dense foods rich in fat and sugar.

Despite the existing evidence, this study makes important contributions. First, by investigating two distinct screen-related behaviors—TV watching time and the frequency of media use during meals. Second, by distinguishing TV watching time from sedentary behavior in general, isolating the specific association with TV watching. Third, by assessing the use of different types of media during meals. The practice of eating while using a cell phone, for example, is a widespread behavior among young people but has been little explored in the literature. Methodologically, this study makes important contributions by using the Nova classification to define UPF, filling a gap in studies on this association in pediatric populations^23^. The exclusive focus on adolescents also allows for an in-depth analysis of this group, whose specific characteristics are often overlooked when they are grouped with children.

One of the main mechanisms explaining the increase in food intake resulting from screen use is constant exposure to the marketing of ultra-processed foods^24^. Although adolescents have more developed cognitive abilities than children, they are particularly susceptible to food marketing due to neurological, hormonal, and social developmental factors^25^. One study demonstrated that TV marketing of UPF strongly appeals to adolescents, directly influencing their preferences and consumption patterns^26^. Companies also use social media to reach this population, employing common tactics such as promoting UPF and using celebrities and influencers^27^.

In this context, digital media form a complex digital food environment, in which the marketing of UPF and fast-food chains is amplified by immersive, integrated, and personalized techniques^25,28^. Digital marketing relies on native advertising and on influencers and content creators, who integrate ads into their posts, presenting them as if they were their genuine opinions, experiences, or preferences^25^. In addition, behavioral advertising, based on the processing of personal data, uses recommendation algorithms to tailor the content or products displayed to each user’s specific interests and behaviors. This personalized targeting increases engagement on platforms and screen time^29^, keeping adolescents constantly exposed to inappropriate food-related stimuli, even during mealtimes.

In the Brazilian context, exposure to UPF marketing is widespread, with nearly 90% of the most-advertised brands on TV in 2018 belonging to this segment and having a strong presence on social media^30^. Given that a large proportion of the adolescents participating in this study watched TV for excessive amounts of time (> 2 h/day)^31^ and regularly used screens during meals, exposure to this marketing, which often employs abusive and deceptive techniques^32^, is inevitable. Given this, there is an urgent need to strengthen current regulations to protect this population^33^. The government must take the lead in implementing comprehensive and mandatory regulations covering all forms of marketing^25^. Additionally, parents and educators need to limit screen time and guide adolescents to develop a critical perspective on these messages to reduce exposure^17^.

Another reason for the link between screen time and UPF consumption is *“*mindless eating”^23^. Distractions during meals, such as watching TV or using electronic devices, compromise attention to food, which can lead to impulsive food choices and a loss of awareness of hunger and satiety^16,34^. In a qualitative study^35^, adolescents reported that the convenience and practicality of processed foods facilitated their consumption while using screens. They described compulsive and automatic eating, associating screen time with a loss of control over satiety, especially when they were alone.

Encouraging mindful eating can promote healthier eating behaviors and, consequently, improve the quality of adolescents’ diets^33^. The Guia Alimentar para a População Brasileira^17^ supports this premise by recommending that meals be eaten mindfully, without rushing, and at regular times. The Guia also suggests that, whenever possible, meals be eaten in the company of family and friends, and that related household activities be shared. The decline in the intergenerational transmission of culinary skills is a factor that contributes to the consumption of UPF^36^. Thus, meals eaten at home with the family provide opportunities for adolescents to develop good habits and appreciate the importance of regular meals prepared in appropriate settings^17^.

The emotions generated by screens also influence food consumption. This is relevant because the content frequently accessed on cell phones and televisions during meals, such as movies, news, messages, and social media, often triggers emotions like sadness or euphoria that directly affect eating behavior^24^. Given this, the implementation of parenting strategies is essential^29,37^, since, by strengthening adolescents’ emotional skills, parents help prevent problematic screen use and promote healthier eating habits^24^.

In line with this need, the recent document “Crianças, adolescentes e telas: guia sobre usos de dispositivos digitais” (Children, Adolescents, and Screens: A Guide on the Use of Digital Devices)^29^ sets forth official recommendations for creating a healthier digital environment in Brazil. The Guia highlight that one of the factors that most contributes to the early and excessive use of digital devices by young people is excessive use by adults, who serve as role models and behavioral references. The document recommends that access to electronic devices, apps, and social media during adolescence should be supervised by family members or educators, reiterating that the non-educational use of these technologies in the school setting can be detrimental to the learning process and to child and adolescent development. The Guia also recommends avoiding the use of connected devices, such as cell phones and tablets, during family meals.

Accordingly, the recent Law No. 15,100/2025^38^, which restricts students’ use of electronic devices, represents a step forward in reducing screen time during meals in the school setting, as it preserves recess time from intensive use of digital devices. Since the trend toward restricting cell phone use in schools is recent, there are still few studies analyzing its impacts^29^. However, this regulatory measure has the potential to foster behavioral changes, especially when integrated with Educação Alimentar e Nutricional (Food and Nutrition Education). As recommended by the Base Nacional Comum Curricular (National Common Core Curriculum), Educação Alimentar e Nutricional should integrate contemporary cross-cutting themes, addressing the dimensions of adequate and healthy eating in conjunction with existing curriculum components^39^. In this sense, it is possible to discuss the value of eating mindfully and the importance of shared meals. The integration of restrictions and awareness is essential so that students not only follow the guidelines but also understand their relevance to their own health.

The study revealed significant sociodemographic disparities in excessive screen time, with adolescents in the lowest wealth quintile showing the highest prevalence of TV watching exceeding 2 hours per day and ranking among the highest in daily media use during meals, compared to wealthier strata. This pattern can be attributed to socioeconomic vulnerability, which limits access to leisure activities, safe public spaces, and options for physical activity^40^. This scarcity of resources increases the likelihood that young people will spend more time at home, often unsupervised due to their caregivers’ long work hours, and turn to screens because of a lack of entertainment alternatives. Although high consumption of UPF was lower in this group, greater screen exposure, combined with the widespread availability and low cost of these products, makes them particularly susceptible to a poorer-quality diet.

Considering that Brazil is still a country with a high prevalence of food insecurity—approximately 24.2% of households experienced some degree of food insecurity in 2024^41^—the solution requires public policies that guarantee access to adequate and healthy food at school and at home, combining measures to reduce the cost of fresh foods and the Brazilian basic food basket with taxation and marketing restrictions that encourage a reduction in the consumption of UPF. Black adolescents attending public schools in urban areas also require greater attention because they are more exposed to screens and high consumption of UPF.

No differences were observed in the prevalence of the outcome or exposures according to sex. Furthermore, in the regression models, the interaction between sex and time spent watching TV was tested, as well as between sex and screen use during meals; these interactions were not statistically significant (p > 0.05). For this reason, the results are presented for the total sample, including sex as a covariate, without the need for stratification.

This study has some limitations that should be considered when interpreting the results. First, it is important to note that it is impossible to differentiate the individual association of each type of screen (TV, computer, or cell phone) used during meals, since the PeNSE methodology groups these devices into a single exposure category. Such differentiation would be relevant, given that different screens may be associated with food consumption in distinct ways. Second, although we observed consistent dose-response associations, it is not possible to infer that increased screen time causes higher UPF consumption, due to the study’s cross-sectional design. Additionally, data on consumption and screen use were self-reported, which may have introduced recall bias and social desirability bias.

Finally, the assessment of UPF consumption was simplified using a score based on 13 subgroups, without quantifying the amount or usual frequency. Although the sensitivity of the associations may be influenced by the adopted cutoff point (scores ≥ 7), its selection is supported by empirical validation. Studies^19,42^ have demonstrated that similar UPF consumption scores have good potential to reflect the caloric contribution of these foods in the diet, showing substantial agreement in classifying participants in the top quintile of the distribution when compared to quantitative assessment tools.

It should also be noted that questionnaires based on consumption markers present classification challenges. For example, the question on bread consumption groups sliced bread, hot dog buns, and hamburger buns into a single category. Although homemade versions of these foods are classified as processed, the instrument assumes that such items are predominantly industrially produced (containing emulsifiers and preservatives), which justifies their inclusion as UPF according to the Guia Alimentar para a População Brasileira, even though this may result in some degree of misclassification.

Despite these limitations, the evidence obtained is important for advancing research and clinical practice. The study stands out for its strengths, which ensure the relevance of its results. The large number of participants and broad national representativeness confer high external validity on the study for the Brazilian adolescent population. The investigation of two distinct forms of screen time exposure is a valuable contribution, as it explores growing and understudied behaviors, such as cell phone use at the dinner table. Additionally, the methodological robustness, which includes the use of the Nova food classification and rigorous adjustment for sociodemographic covariates, increases confidence in the associations and reinforces the study’s importance.

The results demonstrate that time spent watching TV and media use during meals are directly associated with high consumption of UPF among Brazilian adolescents. Given the well-known harmful effects of UPF consumption on the present and future health of this age group, it is urgent that public policies be implemented to limit excessive screen time and discourage media use during meals. Additionally, it is essential to promote healthy and sustainable food systems by removing barriers to access to fresh and minimally processed foods.

## References

[1] 1. Monteiro CA, Cannon G, Moubarac JC, Levy RB, Louzada MLC, Jaime PC. The UN decade of nutrition, the NOVA food classification and the trouble with ultra-processing. Public Health Nutr. 2018;21(1):5-17. 10.1017/S1368980017000234 PMC10261019 28322183

[2] 2. Martinez-Steele E, Khandpur N, Batis C, Bes-Rastrollo M, Bonaccio M, Cediel G et al. Best practices for applying the Nova food classification system. Nat Food. 2023;4(6):445-8. 10.1038/s43016-023-00779-w 37264165

[3] 3. Louzada ML, Gabe KT. Classificação de alimentos nova: uma contribuição da epidemiologia brasileira. Rev Bras Epidemiol. 2025;28:e250027. 10.1590/1980-549720250027.2 PMC12129239 40465876

[4] 4. Rauber F, Louzada ML, Martinez Steele E, Rezende LF, Millett C, Monteiro CA et al. Ultra-processed foods and excessive free sugar intake in the UK: a nationally representative cross-sectional study. BMJ Open. 2019;9(10):e027546. 10.1136/bmjopen-2018-027546 PMC6830631 31662351

[5] 5. Baraldi LG, Steele EM, Canella DS, Monteiro CA. Consumption of ultra-processed foods and associated sociodemographic factors in the USA between 2007 and 2012: evidence from a nationally representative cross-sectional study. BMJ Open. 2018;8(3):e020574. 10.1136/bmjopen-2017-020574 PMC5855172 29525772

[6] 6. Cediel G, Reyes M, Louzada ML, Steele EM, Monteiro CA, Corvalán C et al. Ultra-processed foods and added sugars in the chilean diet (2010). Public Health Nutr. 2018;21(1):125-33. 10.1017/S1368980017001161 PMC10260868 28625223

[7] 7. Khandpur N, Cediel G, Obando DA, Jaime PC, Parra DC et al. Sociodemographic factors associated with the consumption of ultra-processed foods in Colombia. Rev Saúde Pública. 2020;54:19. 10.11606/s1518-8787.2020054001176 PMC7006913 32049210

[8] 8. Louzada ML, Cruz GL, Silva KAAN, Grassi AGF, Andrade GC, Rauber F et al. Consumo de alimentos ultraprocessados no Brasil: distribuição e evolução temporal 2008-2018. Rev Saúde Pública. 2023;57:12. 10.11606/s1518-8787.2023057004744 PMC10118420 37075395

[9] 9. Levy RB, Andrade GC, Cruz GL, Rauber F, Louzada ML, Claro RM et al. Três décadas da disponibilidade domiciliar de alimentos segundo a NOVA - Brasil, 1987-2018. Rev Saúde Pública. 2022;56:75. 10.11606/s1518-8787.2022056004570 PMC9388064 35946675

[10] 10. Instituto Brasileiro de Geografia e Estatística. Pesquisa nacional de saúde: 2019. Rio de Janeiro: IBGE; 2020.

[11] 11. Neri D, Steele EM, Khandpur N, Cediel G, Zapata ME, Rauber F et al. Ultraprocessed food consumption and dietary nutrient profiles associated with obesity: a multicountry study of children and adolescents. Obes Rev. 2022;23(Suppl.1):e13387. 10.1111/obr.13387 34889015

[12] 12. Baird S, Choonara S, Azzopardi PS, Banati P, Bessant J, Biermann O et al. A call to action: the second Lancet Commission on adolescent health and wellbeing. Lancet. 2025;405(10493):1945-2022. 10.1016/S0140-6736(25)00503-3 40409329

[13] 13. Rocha LL, Gratão LHA, Carmo AS, Costa ABP, Cunha CD, Oliveira TRPR et al. School type, eating habits, and screen time are associated with ultra-processed food consumption among brazilian adolescents. J Acad Nutr Diet. 2021;121(6):1136-42. 10.1016/j.jand.2020.12.010 33516640

[14] 14. Antoniassi SG, Machado CO, Santos DS, Santos LD, Höfelmann DA. Tempo de tela, qualidade da dieta de adolescentes e características do entorno escolar. Cien Saúde Colet. 2024;29(1):e00022023. 10.1590/1413-81232024291.00022023 38198315

[15] 15. Avery A, Anderson C, McCullough F. Associations between children's diet quality and watching television during meal or snack consumption: a systematic review. Matern Child Nutr. 2017;13(4):e12428. 10.1111/mcn.12428 PMC6866147 28211230

[16] 16. Jensen ML, Dillman Carpentier FR, Corvalán C, Popkin BM, Evenson KR et al. Television viewing and using screens while eating: associations with dietary intake in children and adolescents. Appetite. 2022;168:105670. 10.1016/j.appet.2021.105670 PMC8671257 34478756

[17] 17. Ministério da Saúde (BR). Guia alimentar para a população brasileira. 2a ed. Brasília: Ministério da Saúde; 2014.

[18] 18. Instituto Brasileiro de Geografia e Estatística. Pesquisa nacional de saúde do escolar: 2019. Rio de Janeiro: IBGE; 2021.

[19] 19. Costa CD, Faria FR, Gabe KT, Sattamini IF, Khandpur N, Leite FH et al. Escore nova de consumo de alimentos ultraprocessados: descrição e avaliação de desempenho no Brasil. Rev Saúde Pública. 2021;55:13. 10.11606/s1518-8787.2021055003588 PMC8023324 33886951

[20] 20. Costa CD, Steele EM, Faria FR, Monteiro CA. Score of ultra-processed food consumption and its association with sociodemographic factors in the brazilian national health survey, 2019. Cad Saúde Pública. 2022;38(Suppl.1):e00119421. 10.1590/0102-311X00119421 35544917

[21] 21. Costa CDS, Wendt A, Machado AKF, Ricardo LIC, Werneck AO et al. Ultra-processed food consumption is related to screen time among Brazilian adolescents, adults and older adults. Br J Nutr. 2025;133(1):118-25. 10.1017/S0007114524002848 PMC11793949 39523205

[22] 22. Barros AJ, Victora CG. Indicador econômico para o Brasil baseado no censo demográfico de 2000. Rev Saúde Pública. 2005;39(4):523-9. 10.1590/s0034-89102005000400002 16113899

[23] 23. Martín-Calvo N, Usechi A, Fabios E, Gómez SF, López-Gil JF. Television watching during meals is associated with higher ultra-processed food consumption and higher free sugar intake in childhood. Pediatr Obes. 2024;19(8):e13130. 10.1111/ijpo.13130 38785294

[24] 24. Tabares-Tabares M, Moreno Aznar LA, Aguilera-Cervantes VG, León-Landa E, López-Espinoza A. Screen use during food consumption: does it cause increased food intake? A systematic review. Appetite. 2022;171:105928. 10.1016/j.appet.2022.105928 35051544

[25] 25. World Health Organization. Taking action to protect children from the harmful impact of food marketing: a child rights-based approach. Geneva: WHO; 2023 [cited 2025 Aug 23]. Available from: https://iris.who.int/bitstream/handle/10665/370355/9789240047518-eng.pdf

[26] 26. Harris JL, Sacco SJ, Fleming-Milici F. TV exposure, attitudes about targeted food ads and brands, and unhealthy consumption by adolescents: modeling a hierarchical relationship. Appetite. 2022;169:105804. 10.1016/j.appet.2021.105804 34780811

[27] 27. Kucharczuk AJ, Oliver TL, Dowdell EB. Social media's influence on adolescents' food choices: a mixed studies systematic literature review. Appetite. 2022;168:105765. 10.1016/j.appet.2021.105765 34687823

[28] 28. Ares G, Antúnez L, Alcaire F, Natero V, Otterbring T. Is this advertisement designed to appeal to you? Adolescents' views about Instagram advertisements promoting ultra-processed products. Public Health Nutr. 2024;27(1):e96. 10.1017/S1368980024000533 PMC10993065 38449441

[29] 29. Secretaria de Comunicação Social (BR). Crianças, adolescentes e telas: guia sobre usos de dispositivos digitais. Brasília: SECOM; 2024 [cited 2025 Aug 23]. Available from: https://www.gov.br/secom/pt-br/assuntos/uso-de-telas-por-criancas-e-adolescentes/guia/guia-de-telas_sobre-usos-de-dispositivos-digitais_versaoweb.pdf

[30] 30. Silva JM, Rodrigues MB, Matos JD, Mais LA, Martins AP, Claro RM et al. Use of persuasive strategies in food advertising on television and on social media in Brazil. Prev Med Rep. 2021;24:101520. 10.1016/j.pmedr.2021.101520 PMC8683935 34976602

[31] 31. Tremblay MS, Carson V, Chaput JP, Connor Gorber S, Dinh T, Duggan M et al. Canadian 24-hour movement guidelines for children and youth: an integration of physical activity, sedentary behaviour, and sleep. Appl Physiol Nutr Metab. 2016;41(6 Suppl.3):S311-27. 10.1139/apnm-2016-0151 27306437

[32] 32. Guimarães JS, Mais LA, Leite FH, Horta PM, Santana MO, Martins AP et al. Abusive advertising of food and drink products on Brazilian television. Health Promot Int. 2022;37(2):daab025. 10.1093/heapro/daab025 PMC9053462 34279624

[33] 33. López-Gil JF, Quiroz-Cárdenas F, Montenegro-Espinosa JA, Cisneros-Vásquez E, Miño C, Castillo-Miñaca ME et al. Eating distractions as predictors of ultra-processed food consumption and Mediterranean diet adherence in adolescents. Sci Rep. 2025;15(1):7579. 10.1038/s41598-025-91754-x PMC11880373 40038379

[34] 34. Robinson E, Aveyard P, Daley A, Jolly K, Lewis A, Lycett D et al. Eating attentively: a systematic review and meta-analysis of the effect of food intake memory and awareness on eating. Am J Clin Nutr. 2013;97(4):728-42. 10.3945/ajcn.112.045245 PMC3607652 23446890

[35] 35. Rodríguez-Barniol M, Pujol-Busquets G, Bach-Faig A. Screen time use and ultra-processed food consumption in adolescents: a focus group qualitative study. J Acad Nutr Diet. 2024;124(10):1336-46. 10.1016/j.jand.2024.04.015 38697354

[36] 36. Watanabe JA, Nieto JA, Suarez-Diéguez T, Silva M. Influence of culinary skills on ultraprocessed food consumption and Mediterranean diet adherence: an integrative review. Nutrition. 2024;121:112354. 10.1016/j.nut.2024.112354 38458144

[37] 37. Nagata JM, Paul A, Yen F, Smith-Russack Z, Shao IY, Al-Shoaibi AA et al. Associations between media parenting practices and early adolescent screen use. Pediatr Res. 2025;97(1):403-10. 10.1038/s41390-024-03243-y. PMC11626836 38834780

[38] 38. Brasil. Lei Nº 15.100, de 13 de janeiro de 2025. Dispõe sobre a proibição do uso de aparelhos eletrônicos portáteis pessoais por alunos da educação básica em escolas públicas e privadas. Diário Oficial União. 14 jan 2025.

[39] 39. Brasil. Lei Nº 13.666, de 16 de maio de 2018. Altera a Lei Nº 9.394, de 20 de dezembro de 1996 (Lei de Diretrizes e Bases da Educação Nacional), para incluir o tema transversal da educação alimentar e nutricional no currículo escolar. Diário Oficial União. 17 maio 2018.

[40] 40. Santos DS, Hino AA, Höfelmann DA. Iniquidades do ambiente construído relacionado à atividade física no entorno de escolas públicas de Curitiba, Paraná, Brasil. Cad Saúde Pública. 2019;35(5):e00110218. 10.1590/0102-311X00110218 31166418

[41] 41. Instituto Brasileiro de Geografia e Estatística. Pesquisa nacional por amostra de domicílios contínua: segurança alimentar 2024. Rio de Janeiro: IBGE; 2025.

[42] 42. Costa CD, Santos FS, Gabe KT, Steele EM, Marrocos-Leite FH, Khandpur N et al. Description and performance of two diet quality scores based on the nova classification. Rev Saúde Pública. 2024;58:47. 10.11606/s1518-8787.2024058006470 PMC11548913 39607210

