# Supplementary material for: Screen use and consumption of ultra-processed foods among Brazilian adolescents
Source: Rev Saude Publica. 2026 Sep 9;60:e42. doi: 10.11606/s1518-8787.2026060007360 (PMC13623369; doi:10.11606/s1518-8787.2026060007360)
Supplement: Translation

# Translation: Uso de telas e consumo de alimentos ultraprocessados por adolescentes brasileiros

**Authors:** Maria Eduarda da Costa Andrade, Diôgo Vale, Poliana de Araújo Palmeira

**Affiliations:** I Brasil Universidade Federal da Paraíba. Programa de Pós-Graduação em Ciências da Nutrição. João Pessoa, PB, Brasil; II Brasil Instituto Federal de Educação, Ciência e Tecnologia do Rio Grande do Norte. Jucurutu, RN, Brasil

**Keywords:** Adolescente, Alimentos Ultraprocessados, Tempo de Tela, Vigilância Alimentar e Nutricional, Inquéritos Epidemiológicos

## Abstract

**OBJETIVO:**

Investigar a associação entre o uso de telas, incluindo tempo assistindo à televisão e uso de mídias (televisão, computador e celular) durante as refeições, e o alto consumo de alimentos ultraprocessados entre adolescentes brasileiros.

**MÉTODOS:**

Estudo transversal, de base populacional, que utilizou dados da Pesquisa Nacional de Saúde do Escolar de 2019, analisando uma amostra de 123.761 adolescentes (13 a 17 anos). O consumo de alimentos ultraprocessados foi avaliado por um escore baseado na frequência de consumo de treze subgrupos de alimentos ultraprocessados no dia anterior. O alto consumo foi definido por um escore de sete ou mais, correspondente ao quintil superior. A associação foi investigada por meio de modelos de regressão de Poisson, ajustados para sexo, idade, raça/cor da pele autorreferida, região geográfica, localização (urbana/rural) e dependência administrativa da escola (pública/privada), e índice de riqueza.

**RESULTADOS:**

A prevalência de alto consumo de alimentos ultraprocessados (escores ≥ 7) foi de 16,15% (IC95% 15,46–16,86). Observou-se uma relação de dose-resposta com maiores prevalências de acordo com o aumento do uso de telas, tanto para o tempo assistindo à televisão como para o uso de mídias durante as refeições. Adolescentes que assistiam à televisão > 6 h/dia apresentaram uma prevalência 90% maior de alto consumo de alimentos ultraprocessados (RP_a_ = 1,90; IC95% 1,72–2,11) em comparação com aqueles que não assistiam. A associação foi significativa mesmo na categoria de menor tempo de exposição (até 1 h/dia). De forma similar, adolescentes que usavam mídia todos os dias durante as refeições tiveram uma prevalência 55% maior (RP_a_ = 1,55; IC95% 1,43–1,68).

**CONCLUSÕES:**

Os achados demonstram uma associação direta e significativa entre maior uso de tela e o alto consumo de alimentos ultraprocessados em adolescentes brasileiros. Este estudo reforça a necessidade de intervenções em saúde pública que abordem o tempo de tela excessivo como um fator comportamental chave no consumo de alimentos ultraprocessados nessa população.

## INTRODUÇÃO

Alimentos ultraprocessados (AUP) são formulações industriais ricas em aditivos e substâncias derivadas de alimentos, com pouca ou nenhuma quantidade de alimento *in natura*. Incluem açúcares, óleos, gorduras ou sal, e outros componentes não usuais em preparações culinárias, como óleos hidrogenados e xarope de milho^1^. Dentre os seus ingredientes também estão os aditivos alimentares cosméticos, que modificam as características sensoriais do produto, como aromatizantes, corantes, edulcorantes, espessantes e realçadores de sabor^2,3^.

Globalmente, o consumo de AUP é maior em faixas etárias mais jovens. No Reino Unido e nos Estados Unidos, por exemplo, os AUP contribuem, em média, com 68,0% e 67,0% da ingestão energética diária entre adolescentes, respectivamente^4,5^. Embora o consumo seja comparativamente menor em países de baixa e média renda, no Chile, na Colômbia e no Brasil os adolescentes estão entre os maiores consumidores desses produtos^6^. No Brasil, especificamente, os AUP representaram cerca de 26,5% do total de energia consumida pelos adolescentes entre 2017 e 2018^8^.

As Pesquisas de Orçamentos Familiares indicaram aumentos sistemáticos na compra de todas as principais categorias de AUP pelas famílias brasileiras, acompanhados por uma redução na aquisição de alimentos *in natura* e minimamente processados. Essas tendências de mudança do padrão alimentar, marcadas pelo crescente consumo de AUP, estão alinhadas com a elevação da prevalência de doenças crônicas não transmissíveis, com o excesso de peso e a obesidade na população^9^. Em 2019, 19,4% dos adolescentes brasileiros tinham excesso de peso, com a obesidade afetando 8,0% das meninas e 5,4% dos meninos^10^.

Estudos têm demonstrado uma associação entre o aumento na participação dietética de AUP e uma maior densidade energética, maiores quantidades de açúcares livres e menor teor de fibras na dieta. Esses achados sugerem que o consumo de AUP é um potencial determinante da obesidade em adolescentes. O consumo consistente de AUP ao longo do tempo impacta negativamente o estado nutricional e a composição corporal nesse grupo etário^11^. Diante do limitado progresso na redução das doenças crônicas não transmissíveis entre adolescentes e do aumento daquelas relacionadas à obesidade^12^, é urgente identificar seus principais fatores de risco.

Rocha et al.^13^ constataram, a partir de análise transversal dos dados do Estudo de Riscos Cardiovasculares em Adolescentes de 2013 e 2014, que a contribuição calórica percentual de AUP entre adolescentes brasileiros foi significativamente maior entre aqueles que frequentavam escolas privadas, não consumiam as refeições oferecidas pelas escolas, não tomavam café da manhã regularmente, comiam enquanto assistiam à televisão (TV) todos os dias ou quase todos os dias, e que passavam mais tempo em frente às telas. Em consonância, outro estudo nacional recente^14^ observou associação entre o uso excessivo de TV e videogame e a pior qualidade da dieta de adolescentes, analisada pela frequência semanal de marcadores de consumo alimentar.

O uso excessivo de telas é outro determinante do excesso de peso em adolescentes^12^, atribuído sobretudo a alterações na qualidade da dieta^15^. Até recentemente, a TV era a forma mais comum de tempo de tela entre adolescentes, mas atualmente há um aumento no uso de outros tipos de telas, como computadores e celulares, especialmente durante as refeições. Esses hábitos podem afetar o consumo alimentar e precisam ser melhor investigados^16^. Embora a relação entre os AUP e o tempo de tela tenha sido explorada individualmente, a associação entre tempo de TV, frequência do uso de diferentes mídias durante as refeições e consumo de AUP em adolescentes ainda não foi suficientemente investigada em amostras nacionalmente representativas.

Compreender essa complexa relação entre o consumo de AUP e o tempo de tela é fundamental para o desenvolvimento de estratégias de saúde pública eficazes para a prevenção da obesidade e doenças crônicas. A compreensão é necessária para a promoção da alimentação adequada e saudável e para a garantia do Direito Humano à Alimentação Adequada. Ademais, o Guia Alimentar para a População Brasileira^17^ preconiza o consumo de alimentos *in natura* e minimamente processados, desencorajando os AUP. Nesse cenário, este estudo teve como objetivo investigar a associação entre o tempo assistindo à TV, o uso de mídias durante as refeições e o consumo de AUP em uma amostra nacionalmente representativa de adolescentes brasileiros com idade entre 13 e 17 anos.

## MÉTODOS

### Desenho do Estudo e População

Este estudo transversal foi desenvolvido com dados da Pesquisa Nacional de Saúde do Escolar (PeNSE) 2019, um inquérito de base escolar de abrangência nacional realizado no Brasil pelo Instituto Brasileiro de Geografia e Estatística, em parceria com o Ministério da Saúde e o Ministério da Educação. O objetivo do inquérito é coletar informações sobre a saúde dos adolescentes de 13 a 17 anos, estudantes de escolas públicas e privadas em áreas urbanas e rurais de todo o território nacional^18^.

O plano amostral da PeNSE 2019 utilizou amostragem por conglomerados em dois estágios, envolvendo escolas e turmas, o que permitiu estimar parâmetros populacionais para o Brasil, as Grandes Regiões, as Unidades da Federação, e as capitais dos estados^14^. Um total de 124.620 adolescentes foi incluído. Os dados foram coletados de abril a setembro de 2019 em sala de aula, utilizando questionários autoaplicáveis em dispositivo móvel de coleta, sob supervisão^18^. Após a exclusão de 849 estudantes com dados ausentes para o consumo de AUP, a amostra final destas análises compreendeu 123.761 participantes. Considerando o efeito do desenho amostral, os dados representam 11.735.113 adolescentes brasileiros. Para as análises de associação (modelos de regressão), a amostra analítica foi composta por 101.923 e 101.998 participantes, respectivamente, para as exposições “tempo assistindo à TV” e “uso de mídias durante as refeições”. Essa redução em relação à amostra final deve-se à exclusão de dados faltantes em qualquer uma das variáveis de exposição e covariáveis incluídas nos modelos.

### Variável de Desfecho

O consumo de AUP foi avaliado por meio de um questionário (sim ou não) acerca do consumo de 13 subgrupos de AUP no dia anterior à entrevista. Esses subgrupos incluíram: (1) refrigerante; (2) suco de fruta em caixinha ou lata; (3) refresco em pó; (4) bebida achocolatada; (5) iogurte com sabor; (6) salgadinho de pacote (*chips*) ou biscoito/bolacha salgado/a; (7) biscoito/bolacha doce, biscoito recheado ou bolinho de pacote; (8) chocolate, sorvete, gelatina, *flan* ou outra sobremesa industrializada; (9) salsicha, linguiça, mortadela ou presunto; (10) pão de forma, pão de cachorro-quente ou pão de hambúrguer; (11) margarina; (12) maionese, *ketchup* ou outros molhos industrializados; e (13) macarrão instantâneo (miojo), sopa de pacote, lasanha congelada ou outro prato pronto comprado congelado. Foi criado um escore de consumo de AUP, somando o número de respostas afirmativas, que variou de 0 (não consumiu nenhum subgrupo) a 13 (consumiu todos os subgrupos). O desfecho (alto consumo de AUP) foi definido para escores iguais ou superiores a 7. Este ponto de corte corresponde ao quintil superior (aproximado) da distribuição do escore na amostra e está em consonância com estudos epidemiológicos prévios^19^.

### Variáveis de Exposição

O tempo de TV foi relatado pelos adolescentes, considerando apenas os dias úteis da semana (excluindo sábados, domingos e feriados) e seis categorias de resposta: não assiste, até 1 h/dia, > 1 h–2 h/dia, > 2 h–3 h/dia, > 3 h–6 h/dia e > 6 h/dia.

A frequência do uso de mídias durante as refeições (TV, computador ou celular) foi avaliada por meio de cinco categorias de resposta: nenhum dia, um a dois dias, três a quatro dias, cinco a seis dias e todos os dias.

### Covariáveis

As covariáveis investigadas incluíram as características sociodemográficas dos participantes: sexo (masculino e feminino), idade (13–15 anos, 16–17 anos), raça/cor da pele autorreferida (branca, parda, preta, amarela e indígena), região geográfica (Norte, Nordeste, Sudeste, Sul e Centro-Oeste do Brasil), localização (urbano e rural) e dependência administrativa da escola (pública ou privada). A situação socioeconômica foi avaliada por índice de riqueza adaptado de Barros e Victora^22^, construído a partir da análise de componentes principais (ACP). As variáveis incluídas na ACP foram: posse de celular, computador/notebook e carro; acesso à internet, número de banheiros no domicílio (0, 1, 2 e 3 ou mais); presença de empregado(a) doméstico(a); e nível educacional da mãe (ensino fundamental incompleto, fundamental completo, médio incompleto, médio completo, superior incompleto e superior completo). O primeiro componente principal, que explica a maior proporção da variância total dos dados, foi retido para criar uma medida contínua de riqueza. Essa variável foi categorizada em quintis, com o primeiro quintil (Q1) representando os 20% dos adolescentes com menor riqueza e o quinto quintil (Q5) representando os 20% com maior riqueza.

### Análise de Dados

As características sociodemográficas da população foram descritas por meio de frequências relativas (%) e seus respectivos intervalos de confiança de 95% (IC95%), tanto para a população total quanto estratificadas pelo desfecho (alto consumo de AUP) e pelas exposições (tempo de TV e uso de mídias durante as refeições). Diferenças de prevalência entre as categorias sociodemográficas e as exposições foram avaliadas pela sobreposição dos IC95%, com a não sobreposição indicando significância estatística. Em seguida, descreveu-se a prevalência de alto consumo de AUP (≥ 7 subgrupos) entre os adolescentes, conforme o tempo de TV e a frequência do uso de mídias nas refeições.

Para investigar a associação entre cada exposição (tempo de TV e frequência do uso de mídias nas refeições) e o alto consumo de AUP, foram empregados modelos de regressão de Poisson com variância robusta. Foram estimadas razões de prevalência (RP) brutas e ajustadas, com seus respectivos IC95%. As variáveis de exposição também foram incluídas como variáveis ordinais nos modelos para examinar a relação linear com o alto consumo de AUP. Todos os modelos ajustados foram controlados para potenciais fatores de confusão, incluindo sexo, idade, raça/cor da pele, índice de riqueza, região do país, localização da escola (urbana/rural) e dependência administrativa da escola (pública/privada).

Todas as análises estatísticas foram realizadas no *software* Stata versão 15, aplicando o comando svy para incorporar os fatores de ponderação da PeNSE 2019 e a estrutura amostral complexa em dois estágios, garantindo a representatividade da população estudada. A significância estatística foi definida *a priori* por IC95% que não contivesse o valor um e por p-valor ≤ 0,05.

### Aspectos Éticos

A participação dos estudantes foi voluntária e condicionada ao registro de concordância com o Termo de Consentimento Livre e Esclarecido, apresentado na primeira tela do aplicativo da PeNSE 2019 no dispositivo móvel de coleta. O acesso ao questionário era liberado somente após a confirmação dessa concordância. O inquérito obteve aprovação da Comissão Nacional de Ética em Pesquisa do Conselho Nacional de Saúde (Parecer nº 3.249.268)^18^.

## RESULTADOS

A amostra final foi composta por 123.761 adolescentes brasileiros e a distribuição foi similar por sexo. A maioria dos participantes (64,70%) tinha entre 13 e 15 anos e se autodeclarou negra, ou seja, parda (43,57%) ou preta (13,52%). Mais de um terço da amostra pertencia ao quintil mais pobre do índice de riqueza (31,81%), e a maioria estava em áreas urbanas (92,42%) e frequentava escolas públicas (85,46%) (Tabela 1).

**Tabela 1:** Características sociodemográficas, consumo de alimentos ultraprocessados e uso de telas por adolescentes brasileiros. PeNSE, 2019 (n = 123.761).

Variável	Total	
	%	IC95%
Sexo		
Masculino	49,14	48,34–49,94
Feminino	50,86	50,06–51,66
Faixa etária		
13–15 anos	64,70	62,39–66,95
16–17 anos	35,30	33,05–37,61
Raça/cor da pele		
Branca	36,06	35,21–36,91
Parda	43,57	42,71–44,44
Preta	13,52	12,99–14,06
Amarela	3,73	3,49–3,99
Indígena	3,12	2,89–3,37
Índice de riqueza		
1° (mais pobre)	31,81	30,57–33,07
2°	25,09	24,37–25,82
3°	21,23	20,45–22,03
4°	14,41	13,75–15,09
5° (mais rico)	7,47	7,06–7,89
Região		
Norte	10,81	9,94–11,75
Nordeste	28,40	27,20–29,62
Sudeste	38,76	37,22–40,34
Sul	13,75	12,93–14,62
Centro-Oeste	8,27	7,80–8,78
Localização		
Rural	7,58	6,36–9,01
Urbana	92,42	90,99–93,64
Dependência administrativa de escola		
Pública	85,46	84,67–86,21
Privada	14,54	13,79–15,33
Consumo de AUP		
< 7 subgrupos	83,85	83,14–84,54
≥ 7 subgrupos	16,15	15,46–16,86
Tempo assistindo à TV		
Não assiste	23,21	22,57–23,86
Até 1 h/dia	24,84	24,28–25,41
> 1 h–2 h/dia	15,89	15,47–16,32
> 2 h–3 h/dia	11,00	10,61–11,40
> 3 h–6 h/dia	15,78	15,31–16,26
> 6 h/dia	9,28	8,84–9,73
Uso de mídias durante as refeições		
Nenhum dia	27,91	27,15–28,70
1 a 2 dias	7,95	7,60–8,33
3 a 4 dias	7,21	6,90–7,55
5 a 6 dias	6,07	5,80–6,37
Todos os dias	50,84	49,91–51,78

PeNSE: Pesquisa Nacional de Saúde do Escolar; IC95%: intervalo de confiança de 95%; AUP: alimentos ultraprocessados.

Nota: dados faltantes: raça/cor da pele (n = 2.462), índice de riqueza (n = 19.662), tempo assistindo à TV (n = 216), uso de mídias durante as refeições (n = 85).

A distribuição do escore de consumo de AUP, o qual equivale ao número de subgrupos consumidos no dia anterior à entrevista, variou de 0 a 13. Os escores correspondentes a 2 (11,73%), 3 (16,34%), 4 (18,08%), 5 (16,32%) e 6 (12,31%) foram os mais frequentes; 6,42% apresentaram escore 1 e 2,66% escore 0 (dados não apresentados). A prevalência de alto consumo de AUP, definido como a ingestão de sete ou mais subgrupos (quintil superior aproximado), foi de 16,15% (Tabela 1).

Aproximadamente quatro em cada dez adolescentes apresentaram tempo excessivo de TV (> 2 h/dia), sendo que 9,28% relataram assistir à TV por mais de 6 h/dia. Além disso, cerca de seis em cada dez adolescentes relataram utilizar mídias (TV, computador ou celular) durante as refeições todos os dias (Tabela 1).

A prevalência de alto consumo de AUP variou significativamente entre as características sociodemográficas. As maiores prevalências foram observadas entre adolescentes mais jovens (17,10%), aqueles que se autodeclararam pretos (18,24%), estudantes de escolas públicas (16,54%) e áreas urbanas (16,46%). Além disso, a prevalência de alto consumo de AUP foi menor no quintil de menor riqueza (13,26%) (Tabela 2).

**Tabela 2:** Prevalência (%) e IC95% de escores de consumo de alimentos ultraprocessados iguais ou superiores a sete no dia anterior à entrevista, de acordo com as características sociodemográficas. PeNSE, 2019 (n = 123.761).

Variável	Consumo de sete ou mais subgrupos de AUP	
	%	IC95%
Sexo		
Masculino	16,70	15,97–17,46
Feminino	15,61	14,77–16,48
Faixa etária		
13–15 anos	17,10	16,19–18,05
16–17 anos	14,40	13,50–15,35
Raça/cor da pele		
Branca	16,18	15,23–17,17
Parda	15,68	14,76–16,65
Preta	18,24	16,94–19,61
Amarela	15,09	12,95–17,50
Indígena	15,52	13,67–17,57
Índice de riqueza		
1° (mais pobre)	13,26	12,35–14,22
2°	17,58	16,35–18,88
3°	18,05	16,80–19,37
4°	19,22	17,80–20,73
5° (mais rico)	17,98	16,50–19,56
Região		
Norte	13,84	12,95–14,77
Nordeste	14,48	13,60–15,39
Sudeste	17,58	16,07–19,19
Sul	17,72	16,56–18,95
Centro-Oeste	15,56	14,46–16,72
Localização		
Rural	12,29	10,79–13,96
Urbana	16,46	15,73–17,22
Dependência administrativa da escola		
Pública	16,54	15,74–17,36
Privada	13,85	13,29–14,44

PeNSE: Pesquisa Nacional de Saúde do Escolar; IC95%: intervalo de confiança de 95%; AUP: alimentos ultraprocessados.

Nota: dados faltantes: raça/cor da pele (n = 2.462), índice de riqueza (n = 19.662).

A análise do uso de telas também revelou importantes disparidades sociodemográficas. O tempo excessivo de TV (> 2 h/dia) foi mais prevalente entre adolescentes mais jovens (38,68%) e naqueles do estrato de menor riqueza (40,02%) em comparação com os mais ricos (28,56%). O uso diário de telas durante as refeições foi mais comum em áreas urbanas (52,06% *versus* 35,95% em áreas rurais) e no estrato de menor riqueza (47,32%) do que no mais rico (34,98%) (Tabela 3).

**Tabela 3:** Prevalência de tempo excessivo de TV (> 2 h/dia) (n = 123.545) e de uso diário de mídias durante as refeições (n = 123.676) por adolescentes, segundo características sociodemográficas. PeNSE, 2019.

Variável	Tempo excessivo de TV (> 2 h/dia)		Uso diário de mídias durante as refeições	
	%	IC95%	%	IC95%
Sexo				
Masculino	35,58	34,55–36,63	51,52	50,34–52,70
Feminino	36,51	35,57–37,46	50,19	49,15–51,23
Faixa etária				
13–15 anos	38,68	37,79–39,57	50,18	48,99–51,38
16–17 anos	31,25	30,09–32,43	52,05	50,71–53,40
Raça/cor da pele				
Branca	31,91	30,86–32,97	49,04	47,79–50,28
Parda	37,61	36,52–38,71	50,47	49,26–51,69
Preta	40,85	38,76–42,98	58,09	56,39–59,78
Amarela	39,79	36,88–42,78	47,81	44,80–50,84
Indígena	38,03	35,00–41,15	50,47	46,95–53,99
Índice de riqueza				
1° (mais pobre)	40,02	38,67–41,39	47,93	46,39–49,47
2°	36,73	35,33–38,15	54,65	53,12–56,17
3°	33,34	31,86–34,87	52,88	51,33–54,43
4°	30,98	29,21–32,81	43,71	42,29–45,13
5° (mais rico)	28,56	26,76–30,44	34,98	33,38–36,62
Região				
Norte	38,98	37,61–40,38	38,14	35,82–40,50
Nordeste	35,05	33,83–36,30	48,53	47,21–49,85
Sudeste	37,27	35,76–38,81	57,51	55,69–59,30
Sul	32,86	30,67–35,15	45,20	43,10–47,31
Centro-Oeste	35,26	33,65–36,91	53,57	51,92–55,22
Localização				
Rural	38,53	36,10–41,03	35,95	33,30–38,70
Urbana	35,85	35,02–36,69	52,06	51,08–53,05
Dependência administrativa da escola				
Pública	37,35	36,45–38,26	51,92	50,84–53,00
Privada	28,67	27,66–29,71	44,51	43,58–45,45

PeNSE: Pesquisa Nacional de Saúde do Escolar; IC95%: intervalo de confiança de 95%.

Nota: dados faltantes para tempo excessivo de TV: raça/cor da pele (n = 2.453), índice de riqueza (n = 19.576); dados faltantes para uso diário de mídias durante as refeições: raça/cor da pele (n = 2.453), índice de riqueza (n = 19.576).

O aumento do consumo de AUP entre os adolescentes esteve diretamente ligado ao maior tempo de exposição à TV e ao uso frequente de telas durante as refeições (Figura). A prevalência de alto consumo de AUP foi 10 pontos percentuais maior entre os que assistiam a mais de seis horas de TV (22,74%) do que entre os que não a assistiam (12,54%). Da mesma forma, o uso de telas em todas as refeições elevou a prevalência para 18,87%, quase sete pontos percentuais acima da prevalência observada em adolescentes que não as usavam (11,94%).

**Figura:** Consumo de sete ou mais subgrupos de alimentos ultraprocessados de acordo com o tempo assistindo à televisão (TV) (n = 123.545) e uso de mídias durante as refeições (n = 123.676). PeNSE, 2019.

PeNSE: Pesquisa Nacional de Saúde do Escolar; AUP: alimentos ultraprocessados.

Conforme Tabela 4, os modelos de regressão de Poisson confirmam que tanto o tempo de TV quanto o uso de mídias durante as refeições estão positivamente associados ao alto consumo de AUP, independentemente de todas as variáveis sociodemográficas analisadas. Uma relação dose-resposta foi observada para o tempo assistindo à TV (p de tendência < 0,001). Em comparação com os adolescentes que não assistiam TV, aqueles que assistiam por mais de 6 h/dia apresentaram uma razão de prevalência ajustada (RP_a_) de 1,90 (IC95% 1,72–2,11) para o alto consumo de AUP. A associação manteve-se significativa inclusive para o menor tempo de exposição (até 1h/dia), que apresentou uma RP_a_ de 1,17 (IC95% 1,07–1,29).

**Tabela 4:** Razões de prevalências brutas e ajustadas de consumo de sete ou mais subgrupos de alimentos ultraprocessados, de acordo com o tempo assistindo à TV (n = 101.923) e o uso de mídias durante as refeições (n = 101.998). PeNSE, 2019.

Variável	Consumo de sete ou mais subgrupos de AUP			
	RP	IC95%	RP ajustada^a^	IC95%
Tempo assistindo à TV				
Não assiste	Ref.	Ref.	Ref.	Ref.
Até 1 h/dia	1,14	1,05–1,24	1,17	1,07–1,29
> 1 h–2 h/dia	1,24	1,13–1,36	1,29	1,17–1,44
> 2 h–3 h/dia	1,36	1,24–1,49	1,41	1,27–1,58
> 3 h–6 h/dia	1,63	1,49–1,78	1,63	1,47–1,80
> 6 h/dia	1,81	1,65–1,99	1,90	1,72–2,11
p de tendência			< 0,001	
Uso de mídias durante as refeições				
Nenhum dia	Ref.	Ref.	Ref.	Ref.
1 a 2 dias	1,15	1,02–1,30	1,16	1,02–1,32
3 a 4 dias	1,28	1,14–1,45	1,27	1,11–1,45
5 a 6 dias	1,40	1,22–1,60	1,41	1,22–1,64
Todos os dias	1,58	1,46–1,70	1,55	1,43–1,68
p de tendência			< 0,001	

PeNSE: Pesquisa Nacional de Saúde do Escolar; IC95%: intervalo de confiança de 95%; RP: razão de prevalência; AUP: alimentos ultraprocessados.

Nota: Modelos de regressão de Poisson foram utilizados para avaliar as associações brutas e ajustadas.

^a^ Ajuste para: sexo, faixa etária, raça/cor da pele, índice de riqueza, região, localização e dependência administrativa da escola (pública e privada).

Da mesma forma, o uso de mídias durante as refeições demonstrou uma relação dose-resposta positiva e significativa com o alto consumo de AUP (p de tendência < 0,001). Adolescentes que usavam mídias todos os dias durante as refeições tiveram uma RP_a_ de 1,55 (IC95% 1,43–1,68) para o alto consumo de AUP, em comparação com aqueles que não usavam mídias. As associações foram significativas a partir de um a dois dias por semana (RP_a_ = 1,16; IC95% 1,02–1,32).

## DISCUSSÃO

Este amplo estudo transversal revelou que tanto o tempo assistindo à TV quanto o uso de diferentes tipos de mídia durante as refeições associaram-se diretamente ao alto consumo de AUP no dia anterior à entrevista, com um claro padrão dose-resposta. Os achados evidenciam a consistente relação entre o comportamento de uso de telas e o consumo de AUP por adolescentes.

Os resultados são consistentes com estudos prévios que investigaram a relação entre a exposição a telas e uma pior qualidade da dieta em adolescentes^13,14^. No Brasil, pesquisa recente demonstrou uma associação positiva entre o tempo dedicado à TV e a outras telas com o consumo de AUP^21^. Essa tendência parece ser global, com estudos no Chile^16^ e no Reino Unido^4^reforçando a conexão entre maior tempo de tela e dietas de pior qualidade. Ainda, uma revisão sistemática^15^identificou que o hábito de comer assistindo à TV entre crianças e adolescentes está ligado a uma maior ingestão de alimentos densamente energéticos e ricos em gordura e açúcar.

Apesar das evidências existentes, este estudo avança em pontos importantes. Primeiro, por investigar dois comportamentos relacionados a telas distintos – o tempo de TV e a frequência do uso de mídias durante as refeições. Segundo, por diferenciar o tempo assistindo à TV do comportamento sedentário em geral, isolando a associação específica de assistir à TV. Terceiro, por avaliar o uso de diferentes tipos de mídias durante as refeições. A prática de realizar refeições utilizando o celular, por exemplo, é um comportamento amplamente comum entre os jovens, mas pouco explorado na literatura. Metodologicamente, este estudo traz contribuições importantes ao utilizar a classificação Nova para definir os AUP, preenchendo uma lacuna em estudos sobre essa associação em populações pediátricas^23^. O foco exclusivo em adolescentes permite, ainda, uma análise aprofundada desse grupo, cujas particularidades são frequentemente negligenciadas quando agrupados com crianças.

Um dos principais mecanismos que explicam o aumento da ingestão alimentar em decorrência do uso de telas é a exposição constante ao *marketing* de produtos ultraprocessados^24^. Embora os adolescentes possuam habilidades cognitivas mais desenvolvidas do que as crianças, eles são particularmente suscetíveis ao *marketing* de alimentos devido a fatores de desenvolvimento neurológico, hormonal e social^25^. Um estudo demonstrou que o *marketing* de AUP na TV atrai fortemente os adolescentes, influenciando diretamente suas preferências e padrões de consumo^26^. As empresas também utilizam mídias sociais para atingir essa população, empregando táticas comuns, como a promoção de AUP e o uso de celebridades e influenciadores^27^.

Nesse cenário, as mídias digitais compõem um complexo ambiente alimentar digital, no qual o *marketing* de AUP e de redes de *fast-food* é amplificado por técnicas imersivas, integradas e personalizadas^25,28^. O *marketing* digital apoia-se na publicidade nativa e nos influenciadores e produtores de conteúdo, que integram anúncios às suas publicações, apresentando-os como se fossem sua opinião, experiência ou preferência genuína^25^. Além disso, a publicidade comportamental, fundamentada no tratamento de dados pessoais, utiliza algoritmos de recomendação para adaptar o conteúdo ou os produtos exibidos aos interesses e comportamentos específicos de cada usuário. Esse direcionamento personalizado aumenta o engajamento nas plataformas e o tempo de tela^29^, mantendo os adolescentes em contínua exposição a estímulos alimentares inadequados, inclusive durante o momento das refeições.

No contexto brasileiro, a exposição ao *marketing* de AUP é massiva, com quase 90% das marcas mais anunciadas na TV em 2018 pertencendo a esse segmento e com forte presença nas mídias sociais^30^. Considerando que grande parte dos adolescentes participantes deste estudo assistia à TV por tempo excessivo (> 2 h/dia)^31^ e usava telas durante as refeições regularmente, a exposição a esse *marketing*, que muitas vezes usa técnicas abusivas e enganosas^32^, é inevitável. Diante disso, é urgente fortalecer as regulamentações atuais para proteger essa população^33^. O governo deve liderar a implementação de regulamentações abrangentes e obrigatórias, que cubram todas as formas de *marketing*^25^. Complementarmente, pais e educadores precisam limitar o tempo de tela e orientar os adolescentes a desenvolverem um senso crítico sobre essas mensagens para reduzir a exposição^17^.

Outra razão para a ligação entre tempo de tela e consumo de AUP é o “comer distraído” (*mindless eating*)^23^. Distrações durante as refeições, como assistir à TV ou usar dispositivos eletrônicos, comprometem a atenção à comida, o que pode levar a escolhas alimentares impulsivas e à perda da percepção de fome e saciedade^16,34^. Em um estudo qualitativo^35^, adolescentes relataram que a conveniência e a praticidade dos AUP facilitavam o consumo durante o uso de telas. Eles descreveram um consumo compulsivo e automático, associando a exposição a telas à perda de controle sobre a saciedade, especialmente quando estavam sozinhos.

Incentivar a alimentação consciente pode promover comportamentos alimentares mais saudáveis e, consequentemente, melhorar a qualidade da dieta em adolescentes^33^. O Guia Alimentar para a População Brasileira^17^ corrobora essa premissa ao recomendar que as refeições sejam feitas com atenção, sem pressa e em horários regulares. O Guia também sugere que, sempre que possível, as refeições sejam realizadas em companhia de familiares e amigos, e que as atividades domésticas relacionadas sejam compartilhadas. O enfraquecimento da transmissão de habilidades culinárias entre gerações é um fator que favorece o consumo de AUP^36^. Assim, as refeições feitas em casa e em família são oportunidades para que os adolescentes adquiram bons hábitos e valorizem a importância de refeições regulares, preparadas em ambientes apropriados^17^.

As emoções geradas pelas telas também influenciam o consumo alimentar. Isso é relevante porque o conteúdo frequentemente acessado em celulares e televisores durante as refeições, como filmes, notícias, mensagens e uso de redes sociais, costuma provocar emoções como tristeza ou euforia que afetam diretamente o comportamento alimentar^24^. Diante disso, a implementação de estratégias parentais é fundamental^29,37^, pois, ao fortalecer as habilidades emocionais dos adolescentes, os pais ajudam a prevenir o uso problemático de telas e a promover hábitos alimentares mais saudáveis^24^.

Alinhado a essa necessidade, o recente documento “Crianças, adolescentes e telas: guia sobre usos de dispositivos digitais”^29^ estabelece recomendações oficiais para a construção de um ambiente digital mais saudável no cenário brasileiro. O Guia destaca que um dos fatores que mais contribuem para o uso precoce e excessivo de dispositivos digitais por jovens é o uso excessivo por parte dos adultos, que são modelos e referências de comportamento. O documento preconiza que o acesso a dispositivos eletrônicos, aplicativos e redes sociais na adolescência deve ser mediado por acompanhamento familiar ou de educadores, reiterando que o uso não pedagógico dessas tecnologias no ambiente escolar pode acarretar prejuízos ao processo de aprendizagem e ao desenvolvimento infantojuvenil. O Guia recomenda, ainda, não acessar dispositivos conectados, como celulares e tablets, durante as refeições em família.

Em consonância, a recente Lei nº 15.100/2025^38^, que restringe o uso de eletrônicos por estudantes, representa um avanço para reduzir telas durante as refeições no ambiente escolar, uma vez que preserva o período do recreio do uso intensivo de dispositivos digitais. Visto que a tendência de restrição de celulares em escolas é recente, ainda existem poucos estudos que analisam seus impactos^29^. Contudo, essa medida regulatória possui potencial para subsidiar mudanças comportamentais, sobretudo se articulada com a Educação Alimentar e Nutricional. Conforme preconizado pela Base Nacional Comum Curricular, a Educação Alimentar e Nutricional deve integrar os temas transversais contemporâneos, abordando as dimensões da alimentação adequada e saudável articulada aos componentes curriculares já existentes^39^. Nesse sentido, é possível dialogar sobre a valorização do ato de comer com atenção e a importância da comensalidade. A integração entre a restrição e a conscientização é fundamental para que os estudantes não apenas sigam a norma, mas também compreendam sua relevância para a própria saúde.

O estudo demonstrou importantes disparidades sociodemográficas na exposição excessiva a telas, com adolescentes no quintil de menor riqueza apresentando as maiores prevalências de tempo de TV superior a 2 h/dia e estando entre as maiores prevalências de uso diário de mídias durante as refeições, em comparação aos estratos mais ricos. Esse padrão pode ser atribuído à vulnerabilidade socioeconômica, que limita o acesso a atividades de lazer, espaços públicos seguros e opções para a prática de atividade física^40^. Essa restrição de recursos aumenta a probabilidade de os jovens passarem mais tempo em casa, muitas vezes desacompanhados devido às longas jornadas de trabalho dos responsáveis, recorrendo às telas devido à escassez de alternativas de entretenimento. Embora o alto consumo de AUP tenha sido menor nesse grupo, a maior exposição a telas, somada à ampla disponibilidade e ao baixo custo desses produtos, os torna particularmente suscetíveis a uma dieta de pior qualidade.

Considerando que o Brasil ainda é um país com alta prevalência de insegurança alimentar – cerca de 24,2% dos domicílios apresentavam algum grau de insegurança alimentar em 2024^41^ – a solução exige políticas públicas que garantam o acesso à alimentação adequada e saudável na escola e no domicílio, reunindo ações conjugadas para redução do custo de alimentos *in natura* e da cesta básica brasileira e taxação e restrições de *marketing* que incentivem a redução no consumo de AUP. Adolescentes de raça/cor de pele preta, de escolas públicas e de áreas urbanas também requerem maior atenção por estarem mais expostos às telas e ao alto consumo de AUP.

Não foram observadas diferenças nas prevalências do desfecho e das exposições de acordo com o sexo. Além disso, nos modelos de regressão, testou-se a interação entre o sexo e o tempo assistindo à TV, assim como entre o sexo e o uso de telas durante as refeições, as quais não se mostraram estatisticamente significativas (p > 0,05). Por essa razão, os resultados são apresentados para a amostra total, incluindo o sexo como variável de ajuste, sem a necessidade de estratificação.

Este estudo possui algumas limitações a serem consideradas na interpretação dos resultados. Primeiramente, destaca-se a impossibilidade de diferenciar a associação individual de cada tipo de tela (TV, computador ou celular) utilizada durante as refeições, visto que a metodologia da PeNSE agrupa tais dispositivos em uma única categoria de exposição. Essa diferenciação seria relevante, dado que diferentes telas podem se associar de forma distinta ao consumo alimentar. Em segundo lugar, embora tenhamos observado associações com padrão dose-resposta consistentes, não é possível inferir que o aumento do tempo de tela cause o maior consumo de AUP, devido ao desenho transversal do estudo. Adicionalmente, os dados sobre consumo e uso de telas foram autorrelatados, o que pode ter gerado vieses de recordação e de desejabilidade social.

Por fim, a avaliação do consumo de AUP foi simplificada com um escore baseado em 13 subgrupos, sem quantificar a quantidade ou a frequência habitual. Embora a sensibilidade das associações possa ser influenciada pelo ponto de corte adotado (escores ≥ 7), sua escolha é suportada por validação empírica. Estudos^19,42^ demonstraram que escores de consumo de AUP similares possuem bom potencial para refletir a contribuição calórica desses alimentos na dieta, apresentando concordância substancial na classificação dos participantes no quintil superior da distribuição quando comparados a instrumentos de avaliação quantitativa.

Deve-se considerar também que questionários baseados em marcadores de consumo apresentam desafios de classificação. Por exemplo, a pergunta sobre o consumo de pães agrupa pão de forma, de cachorro-quente e de hambúrguer em uma única categoria. Embora versões artesanais desses alimentos sejam classificadas como processadas, o instrumento pressupõe o perfil majoritariamente industrializado de tais itens (contendo emulsificantes e conservantes), o que justifica sua inclusão como AUP de acordo com o Guia Alimentar para a População Brasileira, ainda que isso possa gerar algum grau de classificação incorreta.

Apesar dessas limitações, as evidências obtidas são importantes para o avanço da pesquisa e da prática clínica. O estudo destaca-se por pontos fortes que garantem a relevância de seus resultados. O grande número de participantes e a ampla representatividade nacional conferem alta validade externa à pesquisa para a população adolescente brasileira. A investigação de duas exposições distintas ao tempo de tela é uma contribuição valiosa, pois explora comportamentos crescentes e pouco estudados, como o uso de celulares à mesa. Adicionalmente, a robustez metodológica, que inclui o uso da classificação Nova de alimentos e o rigoroso ajuste para covariáveis sociodemográficas, aumenta a confiança nas associações e reforça a importância do estudo.

Os resultados demonstram que o tempo assistindo à TV e o uso de mídias durante as refeições estão diretamente associados ao alto consumo de AUP em adolescentes brasileiros. Diante dos conhecidos malefícios do consumo de AUP para a saúde presente e futura desse grupo etário, é urgente que políticas públicas sejam implementadas para limitar o tempo de tela excessivo e desencorajar o uso de mídias durante as refeições. Adicionalmente, é fundamental promover sistemas alimentares saudáveis e sustentáveis, removendo as barreiras de acesso a alimentos *in natura* e minimamente processados.
